# Temporal progression of mediastinal lymphadenopathy in idiopathic pulmonary fibrosis

**DOI:** 10.1183/13993003.00024-2022

**Published:** 2022-04-21

**Authors:** Tim JM Wallis, Eyjólfur Gudmundsson, Katarina Pontoppidan, Nesrin Mogulkoc, Recep Savaş, Ömer Selim Unat, Katharine Vedwan, Sobana Battison, Fiona J Thompson, Christopher J Brereton, Ben G Marshall, Sophie V Fletcher, Luca Richeldi, Joseph Jacob, Mark G Jones

**Affiliations:** 1 National Institute for Health Research Southampton Biomedical Research Centre, University Hospital Southampton, Southampton UK, SO16 6YD; 2 School of Clinical and Experimental Sciences, Faculty of Medicine, University of Southampton UK, SO17 1BJ; 3 Centre for Medical Image Computing, University College London, London, UK WC1E 6JF; 4 Department of Respiratory Medicine, Ege University Hospital, Izmir, Turkey; 5 Department of Radiology, Ege University Hospital, Izmir, Turkey; 6 Department of Cardiothoracic Radiology, University Hospital Southampton, Southampton UK, SO16 6YD; 7 Unità Operativa Complessa di Pneumologia, Università Cattolica del Sacro Cuore, Fondazione Policlinico A. Gemelli, Rome, Italy; 8 UCL Respiratory, University College London, London UK, WC1E 6JF

## To the Editor

Idiopathic pulmonary fibrosis (IPF) is a progressive fibrotic interstitial lung disease (ILD) with limited therapeutic options and poor prognosis.(1) The average life expectancy from diagnosis is 2 to 4 years,(2) however predicting an individual patient disease trajectory is challenging and there are no established clinically available disease biomarkers.(3) Computed tomography (CT) scanning of the chest is an essential part of the diagnostic pathway in IPF with characteristic appearances of usual interstitial pneumonia pattern (UIP) fibrosis,(4) mediastinal lymphadenopathy (MLN) has been described with high prevalence (52-92%) in IPF cohorts.(5–9). Previous studies have identified that the presence of MLN is linked to disease severity and can independently predict reduced survival in IPF(10, 11) and ILD.(9) The presence of MLN has been shown to persist on longitudinal imaging in the majority of patients with IPF who have MLN at baseline.(11) However, whilst temporal progression of MLN has been shown to correlate with worsening CT fibrosis score,(6) whether temporal progression of MLN in IPF confers an additional impact on mortality is unknown. In this study we investigated temporal trends in MLN and their impact on survival in patients with IPF in two independent cohorts.

Consecutive patients with a confirmed specialist multidisciplinary team diagnosis of IPF based on consensus guidelines(4) least two consecutive volumetric inspiratory CT examinations were identified from two medical centers: cohort 1- University Hospital Southampton, UK between 2011 and 2016, and cohort 2 Ege Hospital Izmir, Turkey between 2008 and 2015. Lung biopsies were performed as part of the diagnostic pathway in 22% (n=11), and 26% (n=24) of patients in cohort 1 and 2 respectively. Ethical approval was obtained from the London-Hampstead Research Ethics Committee (Cohort 1) (REC:17/LO/2037) and from the Leeds-East Research Ethics Committee (Cohort 2) (REC:134 20/YH/0120).

CT scans were assessed for MLN independently for each cohort by experienced radiologists blinded to the study outcomes (Cohort 1, KV and SB, Cohort 2, JJ). CTs were reviewed for MLN in accordance with the International Association for the Study of Lung Cancer classifications,(12) with significant MLN defined as a short-axis diameter ≥10 mm (6, 9–11). Subjects with alternative identified causes for MLN (concurrent pulmonary infection, granulomatous disease or malignancy [except basal skin cancer]) were excluded. The rate of temporal progression/regression was calculated by dividing the difference in size between the largest mediastinal lymph node (on either baseline or follow-up scan) and the same node on the other timepoint CT, by the CT interval (years).

Survival analysis was computed using Kaplan-Meier and Cox proportional-hazard models to determine any association between a) the rate of progression of mediastinal lymphadenopathy on a linear scale (mm/year) b) rate of progression of mediastinal lymphadenopathy stratified as ≥1 mm/year or <1 mm/year - a value which would be the smallest reliable measurable interval change. Survival analysis was conducted from time of the follow-up CT to death or censor. Multivariable survival analysis was adjusted for; age, antifibrotic therapy (ever vs. never taken), and either FVC% predicted or DLCO% predicted. P values of <0.05 were deemed significant. Statistical analysis was conducted using *IBM^®^-SPSS^®^ version 26.0.*

51 patients were included in cohort 1 and 92 patients in cohort 2. The mean (standard deviation) CT imaging interval was 2.17 years (1.6) and 1.40 years (0.7) in cohort 1 and 2 respectively. The inter-radiologist agreement for presence on MLN in cohort 1 was 86% Kappa 0.681 Standard error 0.078 p<0.001.

There was no significant difference observed between patients in cohorts 1 and 2 for either; sex (Males, 82% vs. 78% p=0.56), FVC% predicted (87% vs. 79% p=0.07), DLCO% predicted (52% vs. 54% p=0.33), or smoking history (59% vs. 58% p=0.44). Patients in cohort 1 were significantly older (72.5 years vs. 63.9 years p<0.001) and fewer had ever taken antifibrotic therapy (56% vs. 77% p=0.01). Concomitant historic diagnosis of left ventricular dysfunction was present in 5 patients in cohort 1 and 6 patients in cohort 2.

**Figure 1:**
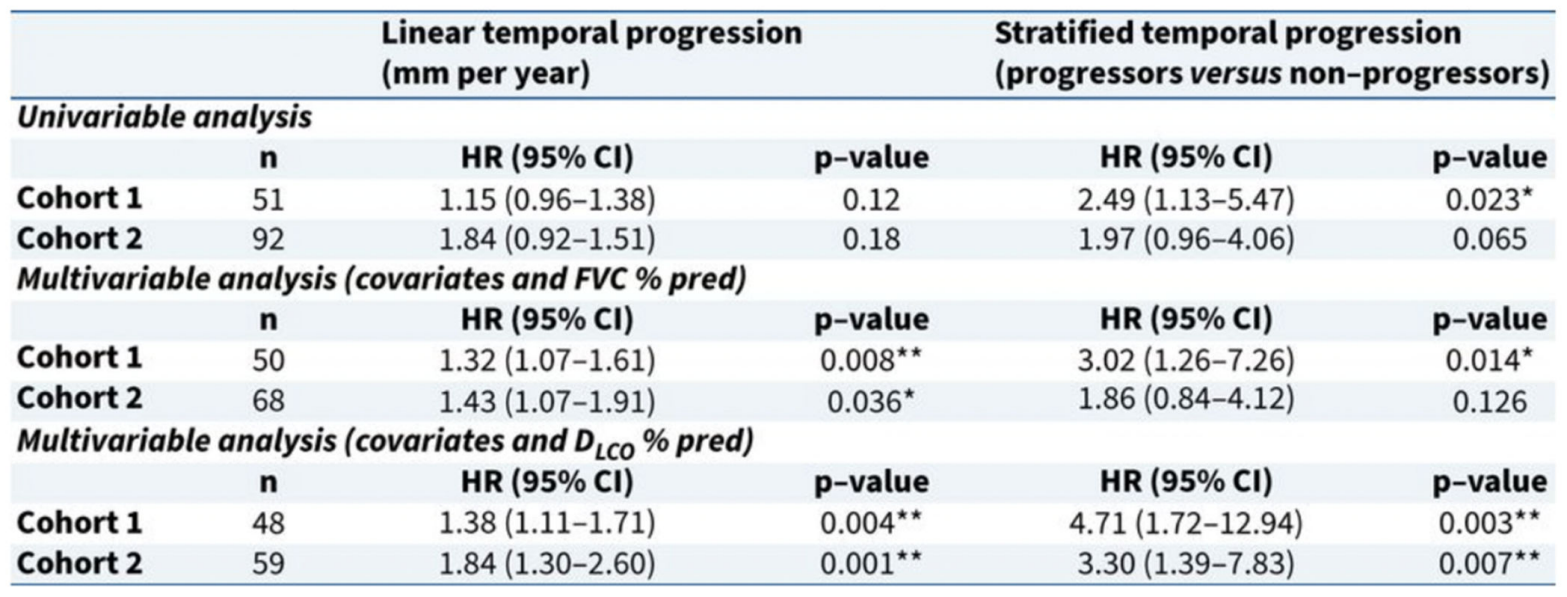

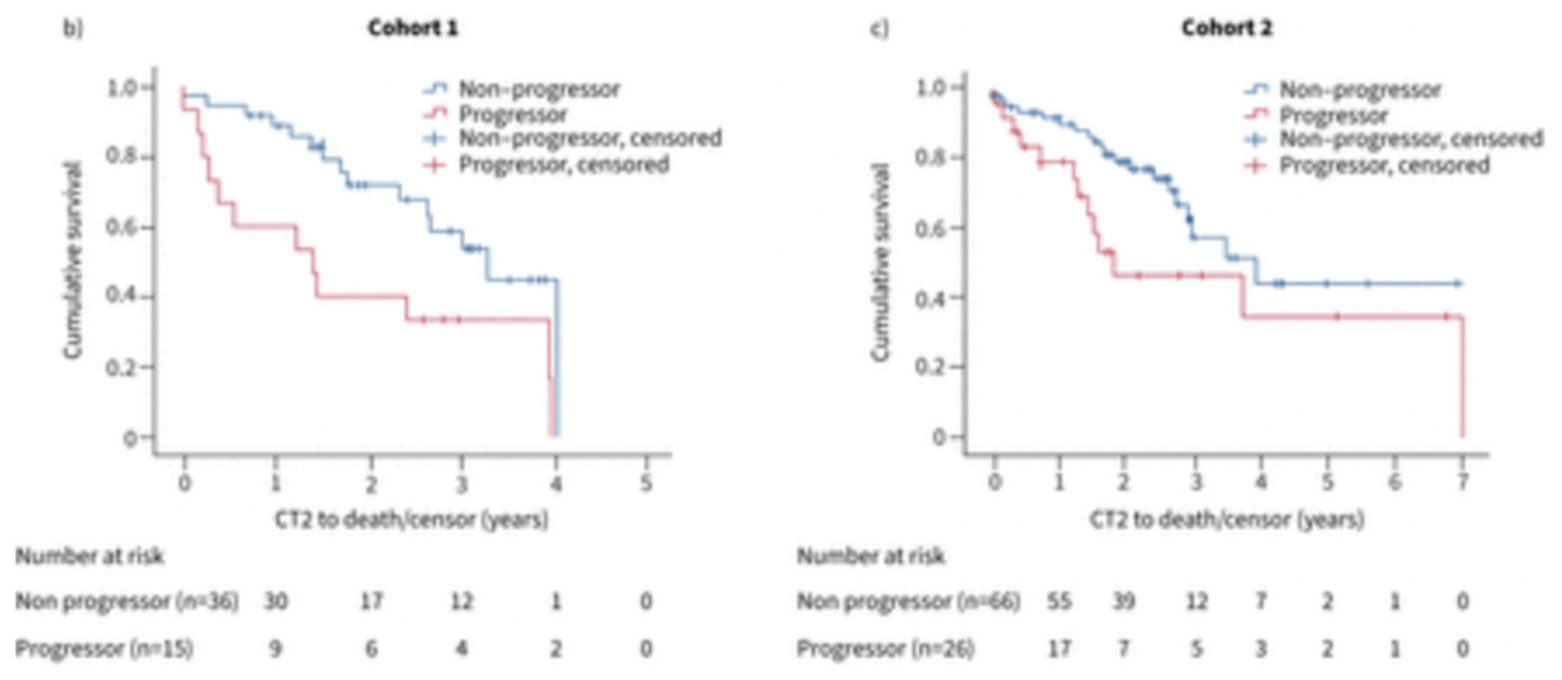
Influence of temporal progression of mediastinal lymphadenopathy (MLN) on survival. ***Figure 1A) Cox Univariable and Multivariable Regression Analyses for influence of temporal progression of MLN on survival***. All multivariable models were adjusted for Age, Antifibrotic therapy (Ever taken vs. never taken), Age, and one of two measures of baseline disease severity, either forced vital capacity percent predicted (FVC% predicted) or diffusion capacity of the lung for carbon monoxide percent predicted (DLCO% predicted). HR-Hazard Ratio, 95%CI-95% Confidence interval, ***Progressors*** - MLN≥10 mm with a ≥1 mm/year increase in size of largest node, ***non-progressors*** - MLN≥10 mm with a <1 mm/year increase in nodal size or no significant MLN on either CT. *p<0.05 **p<0.01. ***Figure 1B and C) Kaplan-Meier cumulative survival curves from Follow-up CT (CT2) to death or censor date (years) for temporal progression of MLN stratified as Progressor vs. Non progressor***. ***Figure 1B)*** Cohort 1 total n=51. Number of deaths per group; Non progressor n=15, Progressor n=12. Log rank p=0.019. ***Figure 1C)*** Cohort 2 n=92. Number of deaths per group; Non progressor n=20 Progressor n=13. Log rank p=0.060.

Significant MLN was identified in 71% (cohort 1) and 84% (cohort 2) of patients at baseline. The incidence of significant MLN increased at follow-up CT and was identified in 88% (cohort 1) and 91% (cohort 2) of patients on either CT scan. At follow-up CT the size of the largest node increased in approximately 50% of patients (55% [cohort 1] and 57% [cohort 2]). The mean unidirectional rate of temporal progression of adenopathy was 1.83 mm/year (cohort 1) and 1.43 mm/year (cohort 2). In univariable analysis the rate of temporal progression of adenopathy (mm/year) trended toward increased mortality risk in both cohorts (Figure 1a). However, in multivariable analysis the rate of temporal change in adenopathy was identified as a significant independent risk factor for mortality in both cohorts (Figure 1a). As a ≥1 mm/year increase would be considered the smallest reliable change, we stratified patients into; ‘***Progressors’*** those with significant MLN and a ≥1 mm/year increase in nodal size, and ‘***Non progressors’*** those with either significant MLN with a <1 mm/year increase in nodal size, or no significant MLN on either scan. Using this stratification within the DLCO% multivariate model identified ‘***Progressors’*** to have a significantly increased mortality risk in both cohorts 1 and 2; Hazard ratio (HR) 4.71 p=0.003 and HR 3.30 p=0.007 respectively (Figure 1a-c). In this study we investigated the temporal progression of MLN in patients with IPF in two independent cohorts. Persistence of adenopathy at follow-up imaging was common and the size of the largest node increased in approximately 50% of patients. We identified that the rate of temporal progression in mediastinal lymphadenopathy was found to predict increased mortality risk. Further stratifying patients using a cut off of a ≥1 mm/year increase in the size of the largest node identified a group with additive poor prognosis.

Despite the observed association between presence of MLN on Chest CTs in IPF patients and increased mortality risk, it remains uncertain whether the development, and the progression in size, of MLN is the driver of, or a reaction to, disease progression.. Proposed mechanisms underlying lymphadenopathy in lung fibrosis include an early response to lung injury facilitating recruitment of inflammatory and fibrotic cells to fibroblastic foci(13, 14) or secondary to local macrophage activation.(15). Consistent with our observation of temporal progression of MLN influencing patient mortality, and the supposition that it is a driver of disease pathology in this group of patients, it has previously been identified that development of MLN between baseline and follow-up CT was significantly associated with worsening fibrosis score in four patients with IPF.(6).

This study is, to our knowledge, the first to assess the impact of temporal progression of MLN on survival in IPF. The strength of our study is the replication of the novel association between temporal progression of MLN and increased mortality risk in two independent cohorts of IPF patients. However, there are a number of methodological limitations including the retrospective analysis, relatively small sample size, and varying intervals of the CT scans performed as part of standard care. Further although CTs were screened for malignancy, it is also important to highlight occult malignancy as a potential confounder

In summary we identify in two independent cohorts of IPF patients that temporal progression of mediastinal lymphadenopathy is frequent and confers an additive and independent increase in mortality risk.
